# Genetic susceptibility to schizophrenia through neuroinflammatory pathways associated with retinal thinness

**DOI:** 10.1038/s44220-025-00414-6

**Published:** 2025-04-21

**Authors:** Finn Rabe, Lukasz Smigielski, Foivos Georgiadis, Nils Kallen, Wolfgang Omlor, Victoria Edkins, Matthias Kirschner, Flurin Cathomas, Edna Grünblatt, Steven Silverstein, Brittany Blose, Daniel Barthelmes, Karen Schaal, Jose Rubio, Todd Lencz, Philipp Homan

**Affiliations:** 1 Department of Adult Psychiatry and Psychotherapy, University of Zurich, Zurich, Switzerland; 2 Department of Child and Adolescent Psychiatry and Psychotherapy, University Hospital of Psychiatry Zurich, University of Zurich, Zurich, Switzerland; 3 Neuroscience Center Zurich, University of Zurich and ETH Zurich, Zurich, Switzerland; 4 Zurich Center for Integrative Human Physiology, University of Zurich, Zurich, Switzerland; 5 Department of Psychiatry, University of Rochester Medical Center, Rochester, NY USA; 6 Department of Ophthalmology, University of Rochester Medical Center, Rochester, NY USA; 7 Department of Neuroscience, University of Rochester Medical Center, Rochester, NY USA; 8 Center for Visual Science, University of Rochester, Rochester, NY USA; 9 Department of Ophthalmology, University Hospital Zurich, University of Zurich, Zurich, Switzerland; 10 Department of Ophthalmology, Inselspital University Hospital Bern, Bern, Switzerland; 11 Institute of Behavioral Science, Feinstein Institutes for Medical Research, Manhasset, NY USA; 12 Division of Psychiatry Research, Zucker Hillside Hospital, Northwell Health, New York, NY USA; 13 Department of Psychiatry, Zucker School of Medicine at Hofstra/Northwell, Hempstead, NY USA

**Keywords:** Schizophrenia, Diagnostic markers, Genetics research

## Abstract

Schizophrenia is associated with structural and functional changes in the central nervous system, including the most distal part of it, the retina. However, the question of whether retinal atrophy is present before individuals develop schizophrenia or is a secondary consequence of the disorder remains unanswered. Here we address this question by examining the association between polygenic risk scores for schizophrenia and retinal morphologies in individuals without a schizophrenia diagnosis. We used population data for 34,939 white British and Irish individuals from the UK Biobank. Our robust regression results show that higher polygenic risk scores for schizophrenia were associated with thinner overall maculae, while controlling for confounding factors (*b* = −0.17, *P* = 0.018). Similarly, we found that greater polygenic risk scores for schizophrenia specific to neuroinflammation gene sets were associated with thinner ganglion cell inner plexiform layers (*b* = −0.10, self-contained *P* = 0.014, competitive *P* = 0.02). These results provide new evidence for genetic factors that could predispose individuals to heightened neuroinflammatory responses. Over time, these responses could contribute to neurodegenerative processes such as retinal thinning.

## Abstract (web summary)

This study explores the relationship between the genetic risk for schizophrenia and retinal thickness, demonstrating that neuroinflammatory pathways are linked to retinal thinning, with C-reactive protein partially mediating this effect. These findings suggest that retinal changes may serve as a potential noninvasive biomarker for schizophrenia risk.

## Main

Individuals with schizophrenia have poor physical health and a reduced life expectancy. Poor physical health is reflected even in the most distal part of the central nervous system, the human retina. The retina is a direct extension of the brain that provides a noninvasive and real-time means of characterizing the neurovascular structure and function of the central nervous system^1^. Recent studies using optical coherence tomography found retinal thinning in individuals with schizophrenia^2–11^. These studies have shown inner retinal atrophy, thinner peripapillary retinal nerve fiber layers and macular ganglion cell and inner plexiform layers, as well as an enlarged cup–disc ratio.

Findings such as these point to a neurobiological substrate of schizophrenia, detectable in this distal part of the central nervous system. However, in all these studies the disorder was already present, and it was thus unclear whether these differences in retinal thickness may have also been detectable at early stages, before the onset of symptoms. In addition, the presence of potentially confounding factors, such as the effects of antipsychotic medication, smoking, lifestyle factors and disease-related changes, can impact retinal health. Such confounders can, in turn, obscure the interpretation of findings, making it difficult to determine whether observed differences in retinal thickness are directly related to the pathophysiology of schizophrenia or are secondary to these confounding factors.

Intriguingly, thinner retinas have been observed not only in patients but also in unaffected first-degree relatives, suggesting a link to genetic susceptibility to schizophrenia^3^. Polygenic risk scores are an alternative to conventional heritability studies. They allow researchers to investigate the genetic underpinnings of the differences in retinal thickness in the context of schizophrenia risk, thus providing a potential understanding of the genetic contributions to retinal atrophy^12^. Polygenic risk scores aggregate the impact of numerous genetic variants throughout the genome and account for a considerable portion of the variance in disease risk^13,14^. The identification of shared genetic influences between retinal structures and schizophrenia^15,16^ further supports the hypothesis that retinal atrophy observed in schizophrenia could reflect underlying genetic susceptibilities. This convergence from optical coherence tomography studies and genetic research may help in the exploration of the ways in which genetic predispositions contribute to the neurodevelopmental and neurodegenerative anomalies in schizophrenia^17^, including retinal alterations.

**Fig. 1:**
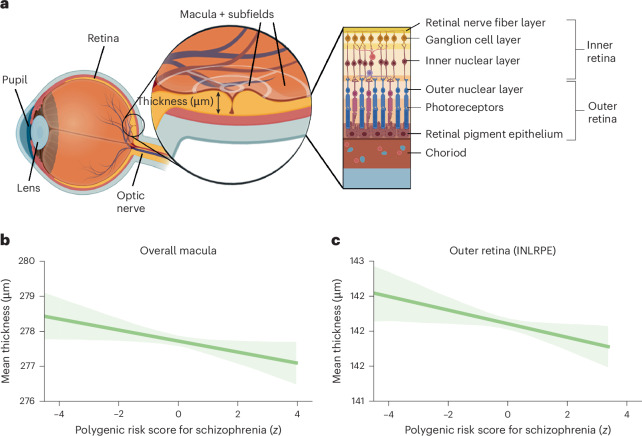
Association between genetic risk for schizophrenia and retinal thickness. **a**, The anatomy of the eye and retina, displayed as a cross section of the eye, and a macular section of the retina. Optical coherence tomography measurements divide the macula into nine subfields, as indicated by the white crosshairs. The macular thickness is measured in micrometers. The structure of the different layers of the inner and outer retina is displayed on the right. **b**,**c**, The association between polygenic risk scores (*z*-scored) for schizophrenia and overall mean macular thickness (in micrometers) (**b**) and outer retinal thickness (**c**) averaged across both eyes. Solid lines represent the regression estimates, while complementary shaded areas correspond to the 95% confidence intervals. INLRPE, inner nuclear layer to the retinal pigment epithelium. Panel **a** was created with BioRender.com.

The present study asked whether thinner retinas are already detectable in healthy individuals with a higher genetic risk for schizophrenia. We focused on the macula, the area with the highest density of neurons in the retina (Fig. 1). Furthermore, we explored gene set specific polygenic risk scores for schizophrenia, which are biological pathways that are related to neurotransmitter regulation, inflammation and microvasculature, all of which may be altered in individuals with schizophrenia^18–21^. Pathway-specific analyses focused on the cumulative genetic risk within specific biological pathways, which may offer insights into the heterogeneity of the disease and its various manifestations^22^. These analyses provide a more nuanced understanding of the genetic architecture of the disorder by exploring whether certain genetic pathways implicated in schizophrenia may also contribute to retinal thinning. This could help in elucidating the biological mechanisms underlying both the psychiatric manifestations and retinal manifestations of schizophrenia. To provide a more comprehensive understanding of how genetic risk affects different retinal structures, we focused on not only the inner retina (including the retinal nerve fiber layer, ganglion cell inner plexiform layer and inner nuclear layer) but also the outer retina (inner nuclear layer–retinal pigment epithelium). If a certain pathway-related genetic risk for schizophrenia is associated with both an increased genetic risk for schizophrenia and inner retinal thickness, this could indicate a shared biological basis that contributes to neurodegenerative processes. In summary, we hypothesized that a higher polygenic risk for schizophrenia would be associated with thinner macular tissue and that this association would be reflected in pathways relevant for schizophrenia.

## Results

### Overall reporting details

**Fig. 2:**
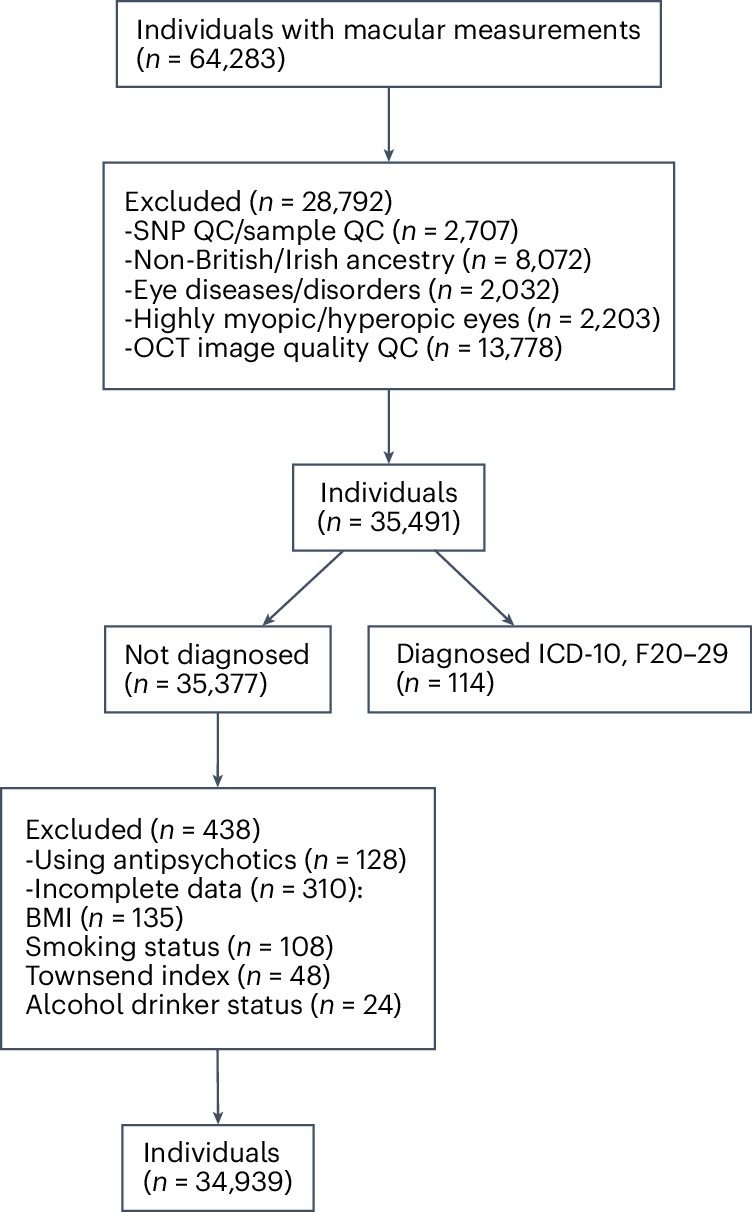
Diagram of inclusion and exclusion of participants in this population. F20–29 categorization includes individuals diagnosed with schizophrenia and schizotypal and delusional disorders. OCT, optical coherence tomography; QC, quality control.

Of the 64,283 individuals recruited with available genetic and retinal data, 34,939 (54.40%) were included for further analysis. Of these participants, 19,070 (54.58%) were female and 15,869 (45.42%) were male (Table 1). We excluded 29,344 individuals owing to various quality control measures related to genetic and optical coherence tomography data, the presence of eye diseases and disorders, the use of antipsychotic medications and diagnoses classified under the International Classification of Diseases tenth revision (ICD-10; F20–F29). For more details, see Fig. 2.

**Table 1:** Key demographic and clinical characteristics of study participants

Characteristic	*N*	Mean (s.d.)
Female	19,070	–
Male	15,869	–
Diabetes mellitus	19,440	–
Hypertension	5,204	–
Current smoker	3,374	–
Previous smoker	12,644	–
Nonsmoker	18,921	–
Current alcohol drinker	32,810	–
Previous alcohol drinker	1,108	–
Nonalcohol drinker	1,021	–
Age (years)	–	56.87 (7.99)
BMI (kg m^−2^)	–	27.23 (4.67)
Townsend index	–	−1.36 (2.81)
Overall macular thickness (right)	–	278.14 (20.37)
Overall macular thickness (left)	–	275.69 (20.37)

The table includes sex, medical conditions (diabetes mellitus and hypertension), smoking status, alcohol consumption habits, age, body mass index (BMI), socioeconomic status (Townsend index) and macular thickness measurements for both eyes. The sample size (*N*) is provided for each category, with the mean and s.d. given for continuous variables where applicable.

Source data

The linear regression models resulting from the analysis of the relationship between polygenic risk scores for schizophrenia and retinal phenotypes displayed heteroscedasticity. This was evidenced by all Breusch–Pagan tests, yielding a chi-squared statistic of *χ*^2^ = 26.93 with *P* < 0.001. This indicates that the variance of the residuals was not constant across the range of values, suggesting that the precision of the regression estimates varies depending on the level of the polygenic risk scores.

### Associations between genetic schizophrenia risk and the retina

The primary dependent variable of the study was the retinal thickness at the macula. We conducted robust regression analyses to examine the relationships between polygenic risk scores for schizophrenia and mean macular thickness. We observed a negative relationship between macular thickness and polygenic risk scores for schizophrenia (*b* = −0.17, 95% confidence interval (CI) −0.31 to −0.03, *P* = 0.018; Fig. 1b). This means that for each 1 s.d. increase in polygenic risk for schizophrenia, the thickness of the macula decreased by 0.17 μm. These robust regression results were similar to those obtained from the imputed dataset analysis (*b* = −0.16, 95% CI −0.30 to −0.02, *P* = 0.028), suggesting the stability of the observed associations across different missing data handling approaches. The detailed association analysis results for the left and right eye and their respective subfields can be found in the Supplementary Information (Supplementary Fig. 1 and Supplementary Table 2). Since the association between inner retinal thickness (specifically retinal nerve fiber layer, ganglion cell inner plexiform layer and inner nuclear layer) has recently been investigated^23^, we extended our analysis to the outer retina. Strikingly, we also found a similar negative association for outer retinal thickness (*b* = −0.10, 95% CI −0.18 to −0.02, *P* = 0.018, *P* value of family-wise error rate (pFWER) of 0.04) (Fig. 1c). The robust regression results for the inner and outer retina are presented in Supplementary Table 1.

### Neuroinflammatory pathway-specific association

Pathway-specific polygenic risk scores are crucial for association analysis with retinal thickness because they allow us to pinpoint specific biological mechanisms underlying the observed retinal changes in individuals with schizophrenia. Intriguingly, we observed that higher polygenic risk scores specific to neuroinflammation in relation to schizophrenia were significantly associated with thinner ganglion cell inner plexiform layers (*b* = −0.10, self-contained *P* = 0.014, competitive *P* = 0.023) (Table 2). We found no statistically significant associations with other gene pathways that could potentially be related to schizophrenia (for more details, see Supplementary Table 4).

**Table 2:** Association between polygenic risk for schizophrenia enriched for multiple gene pathways and inner and outer retinal thickness at a *P* value threshold of 1

GeseaPathwayCode	RNFL	GCIPL	INL	INLRPE
M43559	0.02 (0.03)	−0.02 (0.04)	0.00 (0.02)	−0.02 (0.06)
M6557	0.04 (0.03)	−0.05 (0.04)	0.02 (0.02)	−0.09 (0.06)
M17761	−0.03 (0.03)	−0.06 (0.04)	−0.02 (0.02)	−0.00 (0.06)
M15140	0.04 (0.03)	0.01 (0.04)	0.00 (0.02)	−0.07 (0.06)
M36658	−0.02 (0.03)	0.00 (0.04)	0.01 (0.02)	0.04 (0.06)
M24111	0.03 (0.03)	0.01 (0.04)	−0.01 (0.02)	−0.01 (0.06)
M24927	−0.02 (0.03)	−0.10* (0.04)	−0.01 (0.02)	−0.01 (0.07)
M18933	0.01 (0.03)	−0.05 (0.04)	0.02 (0.02)	−0.09 (0.06)
M25305	−0.03 (0.03)	−0.03 (0.04)	0.02 (0.02)	−0.01 (0.07)
*P < 0.05				

Microvasculature pathways: (1) human phenotype abnormal retinal vascular morphology (M43559) and (2) human phenotype premature coronary artery atherosclerosis (M36658). Inflammatory pathways: (1) Gene Ontology Biological Process (GOBP) acute inflammatory response (M6557), (2) GOBP neuroinflammatory response (M24927), (3) Biocarta transforming growth factor-β (TGFβ) pathway (M18933) and (4) GOBP chronic inflammatory response (M15140). Signaling pathways influencing neuronal development: (1) GOBP WNT signaling pathway involved in midbrain dopaminergic neuron differentiation (M25305), (2) GOBP positive regulation of dopamine receptor signaling pathway (M24111) and (3) ST WNT β-catenin pathway (M17761). Standard errors are presented in brackets. GCIPL, ganglion cell layer to inner plexiform layer; INL, inner nuclear layer; INLRPE, inner nuclear layer to the retinal pigment epithelium; RNFL, retinal nerve fiber layer. Association coefficients are shown in bold to indicate statistical significance. Statistical significance is derived from permutation testing to account for pathway size and linkage disequilibrium structure, so-called self-contained *P* values.

Source data

### The mediating role of C-reactive protein levels

The UK Biobank also provided us with inflammatory marker measurements, which play a pivotal role in managing inflammation. Therefore, we explored the mediating role of these markers in the relationship between neuroinflammatory-enriched polygenic risk scores for schizophrenia and the ganglion cell inner plexiform layer as an outcome variable (Table 3). The mediator model revealed a significant effect of neuroinflammatory-specific polygenic risk scores for schizophrenia on C-reactive protein (CRP; path A coefficient of 0.01), controlling for confounding factors. This positive correlation between neuroinflammation gene enriched polygenic risk scores for schizophrenia and CRP levels suggests that individuals with a higher genetic risk for schizophrenia, particularly in genes related to neuroinflammation, tend to exhibit increased levels of systemic inflammation, as measured by CRP concentrations. Furthermore, the outcome model showed a significant effect of CRP on ganglion cell inner plexiform layer thickness, after accounting for the same covariates, fasting time and neuroinflammatory polygenic risk for schizophrenia. The estimated indirect effect of neuroinflammatory-specific polygenic risk for schizophrenia on the ganglion cell inner plexiform layer through the mediator CRP was −0.001, with a 95% bootstrap CI of −0.003 to −0.0002, suggesting a statistically significant partial mediation effect (*P* = 0.005). This means that part of the negative impact of neuroinflammatory-specific polygenic risk scores for schizophrenia on the ganglion cell inner plexiform layer is mediated by increased CRP levels. More specifically, approximately 1.28% of the total effect of neuroinflammatory-specific polygenic risk scores for schizophrenia on retinal thickness is mediated by CRP levels. This result indicates that, while CRP does play a statistically significant role in mediating the relationship between genetic risk for schizophrenia and retinal thinning, its contribution is relatively small. Most of the effect (about 98.72%) is probably owing to direct effects or other mediating factors not captured by CRP levels alone. For all other inflammatory markers, we did not find such a mediation effect.

**Table 3:** Effects of inflammatory marker on association between neuroinflammatory-specific polygenic schizophrenia risk and ganglion cell inner plexiform layer thickness

	SII	NLR	PLR	LMR	CRP
**Path A**	−1.7	−0.01	−0.2	0	0.01*
	(−4.1 to 0.76)	(−0.01 to 0)	(−0.65 to 0.24)	(−0.02 to 0.01)	(0 to 0.02)
**Path B**	0	−0.01	0	0.02	−0.09**
	(0 to 0)	(−0.05 to 0.04)	(0 to 0)	(0 to 0.04)	(−0.16 to −0.03)
**Total effect**	−0.09**	−0.09**	−0.09**	−0.09**	−0.09**
	(−0.15 to −0.03)	(−0.15 to −0.03)	(−0.15 to −0.03)	(−0.15 to −0.03)	(−0.15 to −0.03)
**Direct effect**	−0.09**	−0.09**	−0.09**	−0.09**	−0.09**
	(−0.15 to −0.03)	(−0.15 to −0.03)	(−0.15 to −0.03)	(−0.15 to −0.03)	(−0.15 to −0.03)
**Indirect effect**	0	0	0	0	–0.001**
	(−0.001 to 0)	(0 to 0)	(−0.001 to 0)	(0 to 0)	(**−0.003 to 0**)

*P < 0.05, **P < 0.01, ***P < 0.001. SII, systemic immune-inflammation index; NLR, neutrophil-to-lymphocyte ratio; PLR, platelet-to- lymphocyte ratio; LMR, lymphocyte-to-monocyte rfatio; Values in brackets represent 95% confidence intervals. Association coefficients of indirect effects are displayed in bold to indicate statistical significance.

## Discussion

In this large observational study in healthy individuals, we found that an increased genetic risk for schizophrenia is associated with lower retinal thickness. This demonstrates that schizophrenia’s genetic risk factors affect not just the brain but also the retina. This result reaffirms the findings of a recent study on the genetic contribution to retinal thickness within the context of schizophrenia risk^23^, in which it was found that, using inner retina-specific analyses, a higher genetic risk for schizophrenia is associated with thinner ganglion cell inner plexiform layers. Our results extend these findings by providing evidence that greater genetic risk for schizophrenia is also associated with thinner outer retinal thickness. This presents a more complete picture on the connection between genetic risk and retinal morphology.

What are the exact mechanisms underlying these retinal alterations in individuals with greater genetic risk for schizophrenia? We provide a potential explanation whereby the expression or activity of a set of genes may trigger neuroinflammatory cascades that affect retinal structure, resulting in measurable changes to the thickness of the ganglion cell inner plexiform layer.

This finding holds great importance as it provides a unique window into the neurobiological underpinnings of schizophrenia, potentially revealing early markers of disease risk and progression.

Inflammatory processes can disrupt the normal functioning of astrocytes, potentially leading to neurotransmitter dysregulation and blood–brain barrier permeability^24,25^. Disruption of the blood–brain barrier, caused by proinflammatory cytokines and chemokines, can exacerbate neuroinflammation by allowing immune cells and potentially harmful substances to enter the brain^24,26^. This can lead to the release of acute-phase proteins, oxidative stress, excitotoxicity and other processes that cause neuronal damage. This in turn affects synaptic functioning, which is critical for normal cognitive processes and has been found to be disrupted in schizophrenia^24,26^. Acute-phase proteins, such as CRP, can also contribute to the progressive apoptosis of photoreceptors^27,28^. This might explain our finding of a partial mediation effect of CRP, suggesting its mediating role alongside other potential candidates, in the association between neuroinflammatory-enriched polygenic risk scores for schizophrenia and retinal thickness. In the case that CRP does serve a mediating role, this would suggest that neuroinflammatory processes, as indexed by CRP levels, could be a key biological pathway through which genetic risk factors for schizophrenia contribute to macular effects. Although a previous study found no association between the levels of inflammatory markers (CRP among others) and retinal thickness in either the schizophrenia or control group^29^, that study was considerably smaller than the present study. It is possible that such an effect can only be detected with substantially more statistical power derived from a large sample size, as in our study.

Intriguingly, the fact that CRP mediation affects the ganglion cell inner plexiform layer suggests that the inflammatory processes associated with schizophrenia risk may have selective effects throughout the retina, presumably targeting amacrine cells in that layer^10^. By examining differential effects on retinal layers, we gained insights into specific cellular and molecular pathways implicated in the disorder, which may lead to the development of more targeted therapeutic interventions.

We also explored more genetic pathways that may contribute to retinal effects in individuals with increasing polygenic risk for schizophrenia. For instance, WNT signaling pathways could play a significant role in the protection of damaged retinal neurons^19,30,31^. They have been shown to have a significant impact on retinal vessel formation and maturation, as well as on the establishment of synaptic structures and neuronal function in the central nervous system^30^. The activation of certain WNT pathways has been shown to reverse the pathological phenotype caused by WNT inhibition in the context of glaucoma^32^. However, in the case of the present study, neither of the WNT signaling pathways showed a significant association with macular thickness changes. This could be because our selection of these pathways was very limited and/or the gene sets of these pathways did not fully capture the polygenic nature of the effects.

In summary, the genetic risk factors for schizophrenia, as represented by the polygenic risk scores, may influence neuroinflammatory pathways involved in the disease. These pathways could, in turn, affect the neural and vascular integrity of the retina, leading to observable retinal changes. While we observed a relationship between polygenic risk scores and retinal layer thickness at the macula, we did not observe any associations between retinal microvasculature gene-based polygenic risk scores for schizophrenia and retinal phenotypes. This could be due to the fact that the genetic architecture of retinal microvasculature traits and schizophrenia risk is complex and again, the selected pathways were not able to capture this relationship. Alternatively, retinal thickness in individuals with high polygenic risk for schizophrenia may also be influenced by other factors, for example, systemic comorbidities such as metabolic dysfunction, certain health behaviors and life-course exposures, which were not evaluated in this study. Future investigations should explore potential gene–environment interactions. For example, given the known effects of smoking on retinal inflammation and ocular health^33,34^, research should examine whether there is an interaction between the polygenic risk score for schizophrenia, smoking and retinal thinning. Such research could provide valuable insights into how lifestyle factors may accelerate neurodegeneration in genetically predisposed individuals^35^.

Several limitations merit comment. The UK Biobank sample is not fully representative of the UK population owing to its low response rate, underrepresentation of younger individuals and socioeconomically deprived areas and lower prevalence of certain health conditions, such as schizophrenia. Our study focused on participants of British or Irish descent, as they constituted the majority of the available data. All of this limits the generalizability of our results to a wider population. Moreover, while robust regression provides advantages in handling outliers and heteroscedasticity, it is crucial to interpret the results in the context of the method’s characteristics. The down weighting of extreme observations might dilute extreme but genuine phenomena. We would also like to note that the temporal disparity in data collection introduced potential unmeasured confounding factors, such as lifestyle changes, medical interventions or environmental influences, which could independently affect inflammatory marker levels or retinal measurements. Finally, the polygenic risk scores’ explanatory power for schizophrenia is modest, and may have limited the detection of small effects. A −0.17 µm change represents only about 0.065% of the average overall macular thickness. This extremely small variation is very unlikely to have an impact on visual acuity, given previous evidence that a much larger change in retinal thickness was associated with only a minimal change in visual acuity^36^.

Our study also had several strengths, including the use of a large, population-based sample from the UK Biobank, which allowed us to avoid confounding factors related to psychotic and retinal abnormalities or antipsychotic use. We also employed robust statistical analyses and complementary techniques to enhance the reliability of our results. Additionally, our extended analyses produced consistent findings across multiple inclusion thresholds for the polygenic risk score. Our study further benefits from the establishment of temporal precedence in the mediation analysis, as inflammatory biomarkers were measured before retinal thickness outcomes, strengthening the causal interpretation of our findings^37^.

In conclusion, our study identified significant associations between polygenic risk scores for schizophrenia, pathway-specific scores and specific retinal layers, offering valuable insights into the genetic contributions to retinal changes in individuals without any diagnosis of schizophrenia. These findings suggest that the retina may serve not only as a ‘window’ to the brain but also as a mirror reflecting the genetic complexities of schizophrenia. They allow us to better distinguish between primary effects of genetic risk and secondary consequences of the disorder. While our results increase confidence that retinal thinning may be associated with core processes in schizophrenia, further research is crucial to establish the specificity and sensitivity of retinal thinning as a reliable indicator of core degenerative processes in the disorder. Future studies should focus on disentangling the complex interplay between genetic predisposition, environmental factors and comorbid conditions that can affect retinal health to determine the true potential of retinal changes as a biomarker for schizophrenia-related processes.

## Methods

### Base data from the 2022 schizophrenia genome-wide association study

The summary statistics base dataset was derived from the latest peer-reviewed genome-wide association study for schizophrenia^13^. We used a file generated with the exclusion of samples from the UK Biobank to assure that the base and discovery files are independent. Following quality control; recommendations^14^, we ensured that the same genome was built with the discovery data (GRCh37/hg19), retained single-nucleotide polymorphisms (SNPs) with minor allele frequency >1% and INFO score >0.8, checked for duplicate SNPs and removed ambiguous SNPs. The final base file included 5,899,135 SNPs.

### Discovery data from the UK Biobank genetic dataset

This study used data from the UK Biobank (application no. 102266). A full description of genotyping and imputation procedures of the UK Biobank data (https://www.ukbiobank.ac.uk/) is provided in the release documentation elsewhere^38^. Briefly, 487,409 blood samples were assayed using two customized tagSNP arrays (the Applied Biosystems UK BiLEVE Axiom Array and the Applied Biosystems UK Biobank Axiom Array; Affymetrix) with 95% shared markers, imputed to the UK10K and 1000 Genome Project phase 3 reference panels, with SHAPEIT3^39^ used for phasing and IMPUTE2^40^ used for imputation. Further data handling and quality control; steps were carried out according to a published processing pipeline^41^. To address population stratification, we retrieved ten genetic principal components from the UK Biobank. Specifically, after SNP extraction and alignment, conversion from bgen to PLINK format and removal of ambiguous SNPs (A/T, C/G: effects with allele frequencies between 0.4 and 0.6), data underwent an SNP-level quality control (minor allele frequency (MAF) <0.005 and INFO score <0.4) and a sample-level quality control; (retaining individuals with missing rate in autosomes <0.02, which were not outliers for genotype missingness or heterozygosity, not genetically related to third-degree relatives, not sex-discordant and of white British or Irish ethnicity according to genetic grouping). We also excluded the 20% of optical coherence tomography images with the poorest image quality and individuals with eye disorders and diseases known to affect the eye, including diabetes related eye diseases, glaucoma, macular degeneration and injury or trauma resulting in loss of vision. We also excluded individuals with highly myopic and hyperopic eyes. Not only did we exclude all individuals that were ‘highly myopic’ according to UK Biobank measurements and criteria^42^, but we also excluded extreme myopic (spherical equivalent (SE) ≥−6 diopters) and hyperopic (SE ≤3 diopters) eyes^43^, as measured by a median SE value for each eye. Finally, individuals with an ICD-10 diagnosis (F20–F29), those who were medicated with antipsychotics and those who had missing data for the variables of interest or covariates were excluded from any further analysis. Table 1 presents details of the sample’s demographic and clinical characteristics. A Little’s missing completely at random test indicated that data were not missing completely at random (*P* < 0.001). Therefore, we used multiple imputation using predictive mean matching to handle missing values and compared regression results between the imputed and complete dataset. In total, 34,939 individuals with matched imaging-genetic data were included in the final analysis (see Fig. 2 for details of participant inclusion and exclusion).

### Polygenic risk scores calculations

In the main analysis, polygenic risk scores for schizophrenia were computed for each individual as a sum of risk alleles weighted by their estimated effect sizes^44^ using the ‘–score’ function in PLINK 2.0 (ref. ^45^). In addition, we generated five polygenic risk scores for schizophrenia for each individual, employing SNPs selected on the basis of their significance in association with the phenotype in the discovery genome-wide association study at nominal *P* value thresholds of 0.01 or less, 0.05, 0.1, 0.5 and 1.00.

### Pathway-based polygenic risk scores and analysis

We prioritized pathways known to be associated with the disease or phenotype of interest based on previous studies^18–21^. Pathway polygenic risk scores were calculated by including only those SNPs that were relevant to the specific pathway under investigation and are also associated with schizophrenia. Pathway polygenic risk scores for schizophrenia were computed using PRSet^14^ in PRSice-2^44^ for nine candidate gene sets selected from the Molecular Signatures Database version v2023.2 (https://www.gsea-msigdb.org/): acute inflammatory response (M6557), TGFβ signaling (M18933), chronic inflammatory response (M15140), positive regulation of dopamine receptor signaling pathway (M24111), WNT signaling pathway involved in midbrain dopaminergic neuron differentiation (M25305), WNT/β-catenin pathways (M17761), neuroinflammatory response (M24927), abnormal retinal vascular morphology (M43559) and premature coronary artery atherosclerosis (M36658). For more details, see ‘Gene pathways from the Molecular Signatures Database (MSigDB) version 7.4 utilized in pathway-based analysis’ in the Supplementary Information. A PRSet *P* value threshold was set at 1 owing to the limited number of SNPs in gene-set polygenic risk scores, potentially not accurately reflecting the entirety of gene sets. Both the self-contained *P* value and the competitive *P* value were obtained. Self-contained methods tested each gene set independently to determine if the genes within the set were associated with the phenotype of interest. This approach does not compare the gene set with the rest of the genome. The level of association reflected by self-contained methods is the degree to which the genes within the pathway are collectively associated with the phenotype, without considering genes outside of the pathway^14^. Competitive methods, on the other hand, compared the level of association between genes within a pathway and the rest of the genes in the genome. This approach tested whether the genes in the pathway are more associated with the phenotype than would be expected by chance, given the level of association observed in genes outside the pathway. Thus, this competitive method reflected signal enrichment by determining if the pathway stood out against the genomic background^14^. Both self-contained and competitive *P* values were calculated within PRSet through 10,000 permutations to generate null distribution curves for *P* values. The regression models consisted of thickness measures for the retinal nerve fiber layer, ganglion cell inner plexiform layer, inner nuclear layer, outer retinal thickness and all covariates. Human GRCh37 genome version was used as the background file.

### Optical coherence tomography protocol and analysis

Optical coherence tomography images were acquired between the years 2009 and 2010 using a spectral domain optical coherence tomography device, with a raster scan protocol with a 6 × 6 mm area centered on the fovea, consisting of 128 B-scans each with 512 A-scans, completed in 3.7 s. Automated analysis of retinal thickness was performed using custom software developed by Topcon Advanced Biomedical Imaging Laboratory, which used dual scale gradient information for rapid segmentation of nine intraretinal boundaries, processing the images in approximately 120 s each. A comprehensive account of the standardized protocol employed for optical coherence tomography acquisition and the subsequent automated analysis of retinal thickness has previously been described^46^. In the current study, we focused on the macula as it contains multiple layers that, based on prior investigations, show thinning in individuals diagnosed with schizophrenia^8,47,48^.

### Assessments of optical coherence tomography data

The normality of the distribution for each retinal phenotype was assessed by visual inspection. However, the tailedness of the distributions for each retinal phenotype data appeared to be skewed by outliers (see the diagonal in Supplementary Fig. 2). We also computed the Pearson correlation coefficients between retinal phenotypes (Supplementary Fig. 2).

### Robust linear regression analysis

To account for potential outliers and heteroscedasticity observed in the retinal phenotype data, robust regression analysis was employed to examine the association between the polygenic risk scores for schizophrenia and macular phenotypes.

Robust linear regression uses M-estimation for robust linear modeling. M-estimators are a broad class of estimators in statistics that generalize maximum likelihood estimators, which are sensitive to outliers and violations of normality assumptions. The estimator used a Huber weight-function to down weight the influence of outliers and heavy-tailed distributions on the estimation of the model parameters^49^. We used the MASS::rlm package in R to conduct this robust analysis in which overall macular thickness was the dependent variable and polygenic risk scores for schizophrenia were the independent variable.

In this study, when reporting *b*, we always refer to the standardized regression coefficient, which represents the estimated change in the dependent variable for a one-unit change in the independent variable.

### Confounding factors

We included a comprehensive set of covariates in a multiple linear regression analysis for studying the association between polygenic risk scores for schizophrenia and macular thickness in isolation. These were age and quadratic age terms, genetic sex, hypertension diagnosed as ICD-10 codes I10–15, diabetes mellitus diagnosed as ICD-10 codes E10–14, alcohol drinker status, BMI, smoking status, Townsend deprivation index, optical coherence tomography image quality, the type of genotyping array used and the first ten genetic principal components. The following rationales were applied for the inclusion of covariates: age is a fundamental factor in the development of diseases, including macular changes and schizophrenia^50^. Various retinal structures are also known to degenerate with age^51,52^. The inclusion of both linear and quadratic terms for age allow the model to capture not just a linear increase or decrease in risk or severity with age, but also any acceleration or deceleration in this trend. Furthermore, biological sex can influence the risk of developing various diseases, their progression and response to treatment^53^, and there is some evidence for sex differences in retinal structural parameters^54^. Hypertension can lead to hypertensive retinopathy, which results in blurred vision, reduced vision or even complete loss of sight if left untreated^55^. Likewise, diabetic retinopathy is a common microvascular complication of diabetes mellitus that can cause vision loss and blindness^56^. Furthermore, alcohol consumption is associated with open-angle glaucoma^57^.

Obesity, as indicated by BMI, is a well-known risk factor for a wide range of health conditions and is associated with retinal layer thickness^50,58^. Similarly, smoking has been linked to an increased risk of various diseases, including those affecting the eyes^53^.

The Townsend deprivation index in the UK Biobank reflects the socioeconomic status, which can influence health outcomes, including those related to ophthalmic health^53^.

The quality of optical coherence tomography images can influence the ability to detect macular changes. Different genotyping arrays can have varying levels of accuracy or might target different sets of genetic variations and thus affect the computation of polygenic risk scores^59^. Likewise, the first ten genetic principal components account for population stratification, which can confound genetic associations. Including them in our models helps to ensure that any associations found are not due to underlying population genetic differences^50^.

### Statistical thresholding

Statistical significance for individual analyses was defined as *P* < 0.05. The pFWER significance threshold using the Holm–Bonferroni method was set at pFWER <0.1.

### Inflammatory biomarkers

The UK Biobank has meticulously recorded an array of inflammatory biomarkers between the years 2006 and 2010. Comprehensive details about their storage and analysis are available at the Biobank showcase. Peripheral blood cell counts, including lymphocyte, monocyte, neutrophil and platelet counts, were obtained using an automated Coulter LH 750 analyzer. The instrument’s differential blood cell count analysis provided calculated values for neutrophil, lymphocyte and monocyte counts, with an operating range of 0.00–900.00 × 10^9^ cells l^−1^. Platelet counts were obtained directly from instrument measurements, with an operating range of 0.00–5000 × 10^9^ cells l^−1^. Using these peripheral blood cell counts, we derived four systemic inflammation markers: systemic immune-inflammation index (SII), neutrophil–lymphocyte ratio (NLR), platelet–lymphocyte ratio (PLR) and lymphocyte–monocyte ratio (LMR). The calculations for these markers were as follows: SII = (neutrophils × platelets)/lymphocytes, NLR = neutrophils/lymphocytes, PLR = platelets/lymphocytes and LMR = lymphocytes/monocytes (ref. ^60^). The measurement of serum CRP levels was carried out using a high- sensitivity immunoturbidimetric method on a Beckman Coulter AU5800 analyzer. In our analysis, we applied a logarithmic transformation to the CRP levels to address their notably skewed distribution, as illustrated in Supplementary Fig. 3.

### Partial effect analyses

To visualize individual phenotypes, we computed the correlation coefficients between the polygenic risk scores for schizophrenia and overall macular thickness, while regressing out all confounding factors. Additionally, we extended this analysis to the combined thickness of the outer retinal layers of the macula.

### Mediation analysis

We further sought to elucidate the mechanisms underlying the association between a pathway-enriched polygenic risk score for schizophrenia, specifically the neuroinflammatory pathway and inner and outer retinal thickness. Moreover, we investigated the potential mediating role of various inflammatory biomarkers in this relationship. The rationale for examining these markers as a mediator is grounded in the evidence linking neuroinflammatory processes to the pathophysiology of schizophrenia^61,62^. Increased levels of inflammatory biomarkers have been associated with increased risk and severity of schizophrenia, suggesting that inflammation may be a biological pathway through which genetic risk factors exert their effects on retinal phenotypes. To assess the mediation effect, we conducted a robust mediation analysis using two models. The mediator model was a robust linear regression that predicted inflammatory levels from neuroinflammatory-enriched polygenic risk for schizophrenia while controlling for the above-mentioned covariates. This model allowed us to estimate the effect of neuroinflammatory polygenic risk for schizophrenia on inflammatory biomarkers (path A). The outcome model was another robust linear regression that predicted ganglion cell inner plexiform layer thickness from both neuroinflammatory-specific polygenic risk for schizophrenia and markers, controlling for the same covariates. This model provided estimates for the direct effect of the neuroinflammatory-specific polygenic risk for schizophrenia on retinal thickness (path C) and the effect of inflammatory biomarkers on retinal thickness, while accounting for the neuroinflammatory-specific polygenic risk for schizophrenia (path B).

The indirect effect (path C′ = path A × path B), representing the mediation effect of inflammatory biomarkers, was calculated as the product of the coefficients from paths A and B. To evaluate the significance of the effect, we employed a bootstrap method with 1,000 resamples, generating empirical 95% CIs for the mediation effect. This nonparametric approach allowed us to infer the robustness of the mediation effect without relying on the assumptions of normality. The *P* value associated with this effect was computed through 95% CI inversion.

Since fasting significantly reduces the number of circulating monocytes^63^, we included fasting time as an additional covariate in this mediation analysis.

### Inclusion and ethics

This study utilized data from the UK Biobank, a large-scale biomedical database and research resource containing in-depth genetic and health information from half a million UK participants. Ethical approval for the use of UK Biobank data was obtained from the North West Multi-center Research Ethics Committee. All participants provided informed consent, and data were anonymized to protect participant privacy. The research team adhered to the UK Biobank’s policies on data access and usage, ensuring compliance with ethical standards and legal requirements. The study design and analysis were conducted with a commitment to inclusivity to ensure broad applicability of the findings.

### Reporting summary

Further information on the research design is available in the Nature Portfolio Reporting Summary linked to this article.

## Supplementary information

Supplementary Information Description of gene pathways and pathway-specific PRS SCZ analysis, Tables 1–4 and Figs. 1–3.

Reporting Summary

## Source data

Source Data Table 1 General polygenic risk scores for schizophrenia for each participant.

Source Data Table 2 Excluded participant based on quality control of genetic data.

## Data Availability

The summary statistics for the schizophrenia genome-wide association study are available at https://pgc.unc.edu/for-researchers/download-results, while the discovery data used in the study are based on the GRCh37/hg19 human genome assembly, which is available via the NCBI Datasets resource at https://www.ncbi.nlm.nih.gov/datasets/genome/GCF_000001405.13/. The rest of the data utilized in this study are available at UK Biobank (http://www.ukbiobank.ac.uk/) and were accessed via application no. 102266.

## Code availability

All data analyses were performed between June 2023 and February 2024 using Python (version 3.9.12) and R (version 4.0.5). The polygenic risk scores were computed using PRSet (version 2.3.5)^14^. The manuscript was produced with the R packages rmarkdown (version 2.21)^64^, MASS (version 7.3.58.4)^65^, sjPlot (version 2.8.14)^66^, represearch (version 0.9; https://github.com/phoman/represearch/), kableExtra (version 1.3.4)^67^, knitr (1.43)^68^, papaja (version 0.1.1)^69^, matplotlib (version 3.7.3)^70^, seaborn (version 0.11.2)^71^ and scipy (version 1.9.1)^72^. All code is available at https://github.com/homanlab/prsoct/ to ensure reproducibility.
