# Supplementary material for: Genetic susceptibility to schizophrenia through neuroinflammatory pathways associated with retinal thinness
Source: Nat Ment Health. 2025 Apr 21;3(5):538–47. doi: 10.1038/s44220-025-00414-6 (PMC12066354; doi:10.1038/s44220-025-00414-6)
Supplement: Supplementary file 1 — Description of gene pathways and pathway-specific PRS SCZ analysis, Tables 1–4 and Figs. 1–3. [file 44220_2025_414_MOESM1_ESM.pdf]

nature mental health

Supplementary information https://doi.org/10.1038/s44220-025-00414-6

Genetic susceptibility to schizophrenia through neuroinflammatory pathways associated with retinal thinness

In the format provided by the authors and unedited

Supplementary Information

Signaling-pathway-specific analyses

A PRSet p-value threshold was set at 1 due to the limited number of SNPs in gene-set polygenic risk scores, potentially not reflecting the entirety of gene sets accurately. Both the self-contained p-value and the competitive p-value were obtained. Self-contained methods tested each gene set independently to determine if the genes within the set were associated with the phenotype of interest. This approach does not compare the gene set to the rest of the genome. The level of association reflected by self-contained methods is the degree to which the genes within the pathway are collectively associated with the phenotype, without considering genes outside the pathway.[14] Competitive methods, on the other hand, compared the level of association between genes within a pathway and the rest of the genes in the genome. This approach tested whether the genes in the pathway are more associated with the phenotype than what would be expected by chance, given the level of association observed in genes outside the pathway. Thus, this competitive method reflected signal enrichment by determining if the pathway stood out against the genomic background.[14] Both self-contained and competitive p-values were calculated within PRSet through 10,000 permutations to generate null distribution curves for p-values. The regression models consisted of thickness measures for retinal nerve fiber layer-, ganglion cell-inner plexiform layer-, inner nuclear layer-, outer retinal thickness and all covariates. Human GRCh37 genome version was used as the background file.

Gene pathways from the Molecular Signatures Database (MSigDB) version 7.4 utilized in pathway-based analysis.
GOBP_NEUROINFLAMMATORY_RESPONSE > Neuroinflammatory response is the nervous system's reaction to various forms of damage, including infection, traumatic brain injury, toxic metabolites, or autoimmunity. [ADCY1 ADCY8 ADORA2A AGER AIF1 APP ATM AZU1 BPGM C1QA C5AR1 CCL3
CD200 CD200R1 CD200R1L CLU CNTF CST7 CTSC CX3CL1 CX3CR1 DAGLA GNAT2
GRN IFNG IFNGR1 IFNGR2 IGF1 IL13 IL18 IL1B IL33 IL4 IL6 ITGAM ITGB1
ITGB2 JAK2 KCNJ8 LARGE1 LDLR LRP1 LRRK2 MAPT MIR128-1 MIR142 MIR181B1
MIR181C MIR195 MIR206 MIR26A1 MMP3 MMP8 MMP9 NAGLU NR1D1 NR3C1 NUPR1
PLCG2 POMGNT1 POMT2 PSEN1 PTGS2 PTPRC SMO SNCA SPHK1 STAP1 SYT11 TAFA3
TLR2 TLR3 TLR6 TLR9 TNF TNFRSF1B TREM2 TRPV1 TTBK1 TYROBP VPS54 ZEB2]

GOBP_CHRONIC_INFLAMMATORY_RESPONSE > This identifier is related to the chronic inflammatory response. A chronic inflammatory response is a prolonged inflammatory reaction that can lead to tissue damage and is involved in various diseases. [ADORA2B CCL5 CXCL13
CYP19A1 FOXP3 IDO1 IL10 IL4 LTA NFKBIZ S100A8 S100A9 SCN11A
THBS1 TNF TNFAIP3 UNC13D VCAM1 VNN1]

BIOCARTA_TGFB_PATHWAY > The TGF beta signaling pathway is a cellular mechanism that regulates various biological processes, including cell growth, differentiation, and apoptosis, and it plays a dual role in inflammation by both promoting and inhibiting inflammatory responses depending on the contex [APC CDH1 CREBBP EP300 MAP2K1 MAP3K7 MAPK3 SKIL SMAD2 SMAD3 SMAD4 SMAD7 TAB1 TGFB1 TGFB2 TGFB3 TGFBR1 TGFBR2 ZFYVE9]

GOBP_ACUTE_INFLAMMATORY_RESPONSE > Inflammation is characterized by a swift, transient, and relatively consistent reaction to acute harm or antigen exposure, marked by fluid, plasma protein, and granulocytic leukocyte accumulations. This acute inflammatory response, which unfolds within minutes or hours, either resolves in a few days or transitions into a chronic inflammatory state. [A2M ACVR1 ADAM8 ADORA1 ADRA2A AHSG ALOX5AP ANO6 APCS APOA2 APOL2 ASH1L ASS1 B4GALT1 BTK C2CD4A C2CD4B C3 CCR7 CD163 CD6 CEBPB CNR1 CREB3L3 CRP CTNNBIP1 DNASE1 DNASE1L3 EDNRB EIF2AK1 ELANE EPO EXT1 F12 F2 F3 F8 FCGR1A FCGR2B FCGR3A FFAR2 FFAR3 FN1 FUT7 GATA3 GSTP1 HAMP HLA-E HP HPR IGHG1 IL1A IL1B IL20RB IL22 IL31RA IL4 IL6 IL6R IL6ST INS ITIH4 KL KLKB1 LBP MBL2 MIR92A1 MRGPRX1 MYLK3 NLRP3 NLRP6 NPY NPY5R NUPR1 OGG1 OPRM1 ORM1 ORM2 OSM OSMR PARK7 PIK3CG PLA2G2D PLSCR1 PRCP PTGER3 PTGES PTGS2 REG3A REG3G RHBDD3 S100A8 SAA1 SAA2 SAA4 SELENOS SERPINA1 SERPINA3 SERPINF2 SIGIRR TACR1 TFR2 TFRC TNF TNFRSF11A TNFSF11 TNFSF4 TREM1

TRPV1 UGT1A1 VCAM1 VNN1 ZP3]

HP_ABNORMAL_RETINAL_VASCULAR_MORPHOLOGY > An identifier suggests a phenotype related to abnormal morphology (structure) of the blood vessels in the retina. [ABCA4 ABCC6 ACVRL1 AGBL5 AHI1
AHR AIPL1 ANTXR1 APOC2 ARHGEF18 ARL2BP ARL3 ARL6 ARV1
ARVCF ASAH1 ATF6 BAZ1B BBS1 BBS2 BBS9 BCL7B BEST1 BUD23
CA4 CAPN5 CC2D2A CCDC28B CCM2 CCND1 CDHR1 CENPF CERKL CFAP410 CFAP418 CHM CLCC1
CLCNKB CLIP2 CLRN1 CNGA1 CNGA3 CNGB1 CNGB3
CNNM4 COL18A1 COL4A1 COMT CRB1 CRX CTC1 CTNNB1 CTSA CYP1B1
CYP27A1 DARS1 DGCR2 DGCR6 DGCR8 DHDDS DHX38 DLK1 DLST DNAJC30 DNMT3A DNMT3B DUX4
DUX4L1 EFEMP1 EIF4H ELN ENG ENPP1 EPAS1
ERCC4 ESS2 ETHE1 EYS F12 FAM161A FGF12 FH FKBP6 FLVCR1 FRG1
FSCN2 FUCA1 FZD4 G6PC1 GALC GDF2 GGCX GLB1 GM2A GNAQ
GNAT2 GP1BB GPIHBP1 GTF2I GTF2IRD1 GTF2IRD2 GUCA1B GUCY2D
HARS1 HEXA HEXB HGSNAT HIRA HK1 HLA-A HSD11B2 IDH3A IDH3B
IFT140 IFT172 IFT43 IFT88 IGFBP7 IKBKG IMPDH1 IMPG1 IMPG2
IVNS1ABP JMJD1C KIAA1549 KIF1B KIZ KLHL7 KRIT1 LAMB2 LCA5
LCK LIMK1 LOXL1 LPL LRAT LRP5 LRRC32 MAK MAX MDH2 MEG3
MERTK METTL27 MKS1 MLXIPL MT-ATP6 MT-CO1 MT-CO3 MT-CYB
MT-ND1 MT-ND2 MT-ND4 MT-ND4L MT-ND5 MT-ND6 MVK MYD88
MYOC NCF1 NDP NDUFS2 NEK1 NEK2 NEU1 NF1 NMNAT1 NOD2
NR2E3 NRL NUS1 OFD1 PAX6 PCARE PCNA PDCD10 PDE6A PDE6B
PDE6C PDE6G PDE6H PIK3CA POMGNT1 PRCD PROM1 PRPF3 PRPF31
PRPF4 PRPF6 PRPF8 PRPH2 PSAP RAX2 RBP3 RDH11 RDH12 RDH5
REEP6 RET RFC2 RGR RHO RLBP1 ROM1 RP1 RP1L1 RP2 RP9 RPE65
RPGR RPGRIP1 RREB1 RTL1 RTTN SAG SCAPER SDHA SDHAF2
SDHB SDHC SDHD SEC24C SELENOI SEMA4A SERPINC1 SETD2
SLC24A1 SLC25A11 SLC37A4 SLC6A6 SLC7A14 SMAD4 SMCHD1
SMPD1 SNRNP200 SPATA7 SSBP1 STN1 STX1A TBL2 TBX1 TLCD3B
TMEM127 TMEM231 TMEM270 TOPORS TREX1 TSPAN12 TTC8 TUB TULP1 UFD1 UNC119 USH2A USP45
VHL VPS37D WDR19 WT1 YME1L1 ZNF408 ZNF513]

HP_PREMATURE_CORONARY_ARTERY_ATHEROSCLEROSIS > This term refers a gene set that is related to the premature development of atherosclerosis in the coronary arteries, which can lead to coronary artery disease (CAD). [ABCA1 ABCG5 ABCG8 ACTA2
APOA1 APOB APOE CEP19 CYP27A1 ERCC6 ERCC8 GPIHBP1 LDLR LDLRAP1 LIPC
LMNA LRP6 MEF2A PCSK9]

ST_WNT_BETA_CATENIN_PATHWAY > This identifier refers to a gene pathway involved in the Wnt/beta-catenin signaling. The Wnt/beta-catenin pathway is crucial for various cellular processes, including development, cell proliferation, and differentiation. [AKT1 AKT2 AKT3
ANKRD6 APC AXIN1 AXIN2 CBY1 CER1 CSNK1A1 CTNNB1 CXXC4 DACT1
DKK1 DKK2 DKK3 DKK4 DVL1 FRAT1 FSTL1 GSK3A GSK3B LRP1 MVP NKD1 NKD2 PIN1 PSEN1 PTPRA
RPSA SENP2 SFRP1 TSHB WIF1]

GOBP_WNT_SIGNALING_PATHWAY_INVOLVED_IN_MIDBRAIN_DOPAMINERGIC_NEURON_DIFFERENTIATION > This identifier contains genes that are related to the role of the Wnt signaling pathway in the differentiation of dopaminergic neurons in the midbrain. This process is important for the development of the nervous system and is particularly relevant to the study of diseases like schizophrenia and Parkinson's disease, where dopaminergic neurons are affected. [CSNK1D CSNK1E CTNNB1 FZD1 LRP6 RYK WNT1 WNT2 WNT3 WNT3A WNT5A WNT9B]

GOBP_POSITIVE_REGULATION_OF_DOPAMINE_RECEPTOR_SIGNALING_PATHWAY > This identifier con-

tains genes responsible for a biological process that positively regulates the signaling pathway of dopamine receptors. Dopamine receptors are critical for many neurological processes, and their signaling pathways are important in the context of neurological diseases, e.g. schizophrenia and Parkinsons's disease. [CAV2 DRD3 LRRK2 PRMT5 VPS35]

Supplementary Tables

Table S1
Robust regression results for inner and outer retina

Phenotype	Coef	CI(low)	CI(high)	SE	BPstat	BPp	F	p
RNFL mean	-0.017	-0.062	0.028	0.02297	66.05	p < .001	0.53	0.46635
GC IPL mean	-0.087	-0.149	-0.026	0.03134	28.37	p = 0.164	7.76	0.00534
INL mean	-0.004	-0.03	0.021	0.0131	49.26	p < .001	0.11	0.73716
INL RPE mean	-0.101	-0.184	-0.018	0.04242	53.87	p < .001	5.64	0.01755

Table S2

Association between polygenic risk for schizophrenia and macular subfields

Macular subfield	hm	Coef	CI(low)	CI(high)	SE	BPstat	BPp	F	p	pFWER	Groups
Inner Inferior left	left	-0.0090831	-0.016	-0.002	0.00351	67.95	p < .001	6.69	0.00971	0.09710	Inner Inferior
Inner Inferior right	right	-0.0111756	-0.018	-0.004	0.00346	53.37	p < .001	10.39	0.00127	0.02032	Inner Inferior
Outer Inferior left	left	-0.0022769	-0.01	0.005	0.00382	59.74	p < .001	0.36	0.55125	1.00000	Outer Inferior
Outer Inferior right	right	-0.0035174	-0.011	0.004	0.00389	47.46	p = 0.001	0.82	0.36596	1.00000	Outer Inferior
Inner Nasal left	left	-0.0108702	-0.018	-0.004	0.00356	59.65	p < .001	9.30	0.00229	0.03435	Inner Nasal
Inner Nasal right	right	-0.0128938	-0.02	-0.006	0.00365	48.59	p < .001	12.43	0.00042	0.00756	Inner Nasal
Outer Nasal left	left	-0.0074260	-0.015	0	0.00394	47.12	p = 0.001	3.55	0.05948	0.41636	Outer Nasal
Outer Nasal right	right	-0.0114548	-0.019	-0.004	0.00393	43.29	p = 0.004	8.48	0.00360	0.04320	Outer Nasal
Inner Superior left	left	-0.0102455	-0.017	-0.004	0.0034	68.31	p < .001	9.07	0.00259	0.03435	Inner Superior
Inner Superior right	right	-0.0117573	-0.019	-0.005	0.00353	54.37	p < .001	11.07	0.00088	0.01496	Inner Superior
Outer Superior left	left	-0.0100111	-0.017	-0.003	0.00366	57.77	p < .001	7.48	0.00624	0.06864	Outer Superior
Outer Superior right	right	-0.0108973	-0.018	-0.004	0.00359	54.48	p < .001	9.20	0.00243	0.03435	Outer Superior
Inner Temporal left	left	-0.0084811	-0.015	-0.002	0.00339	71.5	p < .001	6.26	0.01234	0.11106	Inner Temporal
Inner Temporal right	right	-0.0082580	-0.015	-0.001	0.00346	51.55	p < .001	5.70	0.01696	0.13568	Inner Temporal
Outer Temporal left	left	-0.0067490	-0.014	0	0.00365	44.36	p = 0.003	3.42	0.06450	0.41636	Outer Temporal
Outer Temporal right	right	-0.0050174	-0.013	0.003	0.00384	46.66	p = 0.002	1.71	0.19096	0.95480	Outer Temporal
Central left	left	-0.0032214	-0.012	0.005	0.00433	65.13	p < .001	0.55	0.45765	1.00000	Central
Central right	right	-0.0040272	-0.013	0.005	0.00448	66.62	p < .001	0.81	0.36926	1.00000	Central

Table S3
Association between polygenic risk for schizophrenia (PRSSZ) at different p-value thresholds and mean macular thickness

PRS	Phenotype	Coef	CI(low)	CI(high)	SE	F	p	pFWER
PRSSZ	Macula mean	-0.17	-0.31	-0.029	0.07159	5.59	0.0181	0.0181
PRSSZ 0.001	Macula mean	-0.10	-0.238	0.046	0.07259	1.75	0.1857	0.1857
PRSSZ 0.05	Macula mean	-0.18	-0.345	-0.024	0.08199	5.06	0.0244	0.0244
PRSSZ 0.1	Macula mean	-0.16	-0.325	0.007	0.08454	3.53	0.0601	0.0601
PRSSZ 0.2	Macula mean	-0.16	-0.329	0.003	0.08468	3.70	0.0544	0.0544
PRSSZ 0.3	Macula mean	-0.15	-0.322	0.012	0.08538	3.28	0.0699	0.0699
PRSSZ 0.4	Macula mean	-0.16	-0.322	0.011	0.08512	3.33	0.068	0.0680
PRSSZ 0.5	Macula mean	-0.15	-0.313	0.02	0.08501	2.98	0.0846	0.0846
PRSSZ 1	Macula mean	-0.15	-0.317	0.016	0.08488	3.13	0.0769	0.0769

Table S4

Permutation test regression between polygenic risk for schizophrenia enriched for multiple gene pathways and inner vs. outer retinal thickness at p-value threshold 1 using PRSet.

Pathway	GeseaPathwayCode	Num_SNP	Phenotype	Estimate	SE	selfcontained.p	competitive.p	Groups	CIhigh	CIlow
ABNOVAS PRS	M43559	2267	GCIPL	-0.0216670	0.0370876	0.5590830	0.5983400	Microvasculature	0.051	-0.094
ABNOVAS PRS	M43559	2267	INL	0.0036801	0.0163518	0.8219350	0.8336170	Microvasculature	0.036	-0.028
ABNOVAS PRS	M43559	2267	INLRPE	-0.0175006	0.0629268	0.7809300	0.7873210	Microvasculature	0.106	-0.141
ABNOVAS PRS	M43559	2267	RNFL	0.0201864	0.0261130	0.4395030	0.4640540	Microvasculature	0.071	-0.031
ACUTEINFLAM PRS	M6557	816	GCIPL	-0.0507433	0.0371047	0.1714560	0.2003800	Inflammation	0.022	-0.123
ACUTEINFLAM PRS	M6557	816	INL	0.0153199	0.0163595	0.3490450	0.3770620	Inflammation	0.047	-0.017
ACUTEINFLAM PRS	M6557	816	INLRPE	-0.0901261	0.0629553	0.1522710	0.1643840	Inflammation	0.033	-0.214
ACUTEINFLAM PRS	M6557	816	RNFL	0.0366421	0.0261250	0.1607560	0.1784820	Inflammation	0.088	-0.015
CATENIN PRS	M17761	342	GCIPL	-0.0626347	0.0368550	0.0892364	0.1145890	Developmental	0.010	-0.135
CATENIN PRS	M17761	342	INL	-0.0180391	0.0162495	0.2669520	0.2876710	Developmental	0.014	-0.050
CATENIN PRS	M17761	342	INLRPE	-0.0008908	0.0625345	0.9886350	0.9900010	Developmental	0.122	-0.123
CATENIN PRS	M17761	342	RNFL	-0.0342921	0.0259497	0.1863500	0.2033800	Developmental	0.017	-0.085
CHROINFLAM PRS	M15140	137	GCIPL	0.0135218	0.0374524	0.7180730	0.7288270	Inflammation	0.087	-0.060
CHROINFLAM PRS	M15140	137	INL	0.0046204	0.0165125	0.7796230	0.7938210	Inflammation	0.037	-0.028
CHROINFLAM PRS	M15140	137	INLRPE	-0.0737256	0.0635442	0.2459660	0.2697730	Inflammation	0.051	-0.198
CHROINFLAM PRS	M15140	137	RNFL	0.0393136	0.0263690	0.1359980	0.1502850	Inflammation	0.091	-0.012
CORART PRS	M36658	244	GCIPL	0.0043803	0.0372143	0.9063020	0.9100090	Microvasculature	0.077	-0.069
CORART PRS	M36658	244	INL	0.0079957	0.0164075	0.6260350	0.6457350	Microvasculature	0.040	-0.024
CORART PRS	M36658	244	INLRPE	0.0401633	0.0631410	0.5247230	0.5439460	Microvasculature	0.164	-0.084
CORART PRS	M36658	244	RNFL	-0.0164771	0.0262021	0.5294540	0.5418460	Microvasculature	0.035	-0.068
DOPPOSREG PRS	M24111	69	GCIPL	0.0124878	0.0381142	0.7431860	0.7448260	Developmental	0.087	-0.062
DOPPOSREG PRS	M24111	69	INL	-0.0061939	0.0168043	0.7124350	0.7360260	Developmental	0.027	-0.039
DOPPOSREG PRS	M24111	69	INLRPE	-0.0103033	0.0646684	0.8734130	0.8795120	Developmental	0.116	-0.137
DOPPOSREG PRS	M24111	69	RNFL	0.0300539	0.0268354	0.2627500	0.2787720	Developmental	0.083	-0.023
NEUROINFLAM PRS	M24927	751	GCIPL	-0.0958475	0.0391102	0.0142630	0.0230977	Inflammation	-0.019	-0.173
NEUROINFLAM PRS	M24927	751	INL	-0.0061076	0.0172450	0.7232160	0.7412260	Inflammation	0.028	-0.040
NEUROINFLAM PRS	M24927	751	INLRPE	-0.0093058	0.0663644	0.8884850	0.8903110	Inflammation	0.121	-0.139
NEUROINFLAM PRS	M24927	751	RNFL	-0.0197093	0.0275395	0.4741970	0.4941510	Inflammation	0.034	-0.074
TGFB PRS	M18933	816	GCIPL	-0.0507433	0.0371047	0.1714560	0.2043800	Inflammation	0.022	-0.123
TGFB PRS	M18933	816	INL	0.0153199	0.0163595	0.3490450	0.3800620	Inflammation	0.047	-0.017
TGFB PRS	M18933	816	INLRPE	-0.0901261	0.0629553	0.1522710	0.1700830	Inflammation	0.033	-0.214
TGFB PRS	M18933	247	RNFL	0.0100906	0.0267023	0.7055140	0.7214280	Inflammation	0.062	-0.042
WNT PRS	M25305	107	GCIPL	-0.0303761	0.0386493	0.4319080	0.4606540	Developmental	0.045	-0.106
WNT PRS	M25305	107	INL	0.0232558	0.0170399	0.1723300	0.1992800	Developmental	0.057	-0.010
WNT PRS	M25305	107	INLRPE	-0.0128762	0.0655768	0.8443340	0.8482150	Developmental	0.116	-0.141
WNT PRS	M25305	107	RNFL	-0.0339460	0.0272122	0.2122400	0.2231780	Developmental	0.019	-0.087

Supplementary Figures

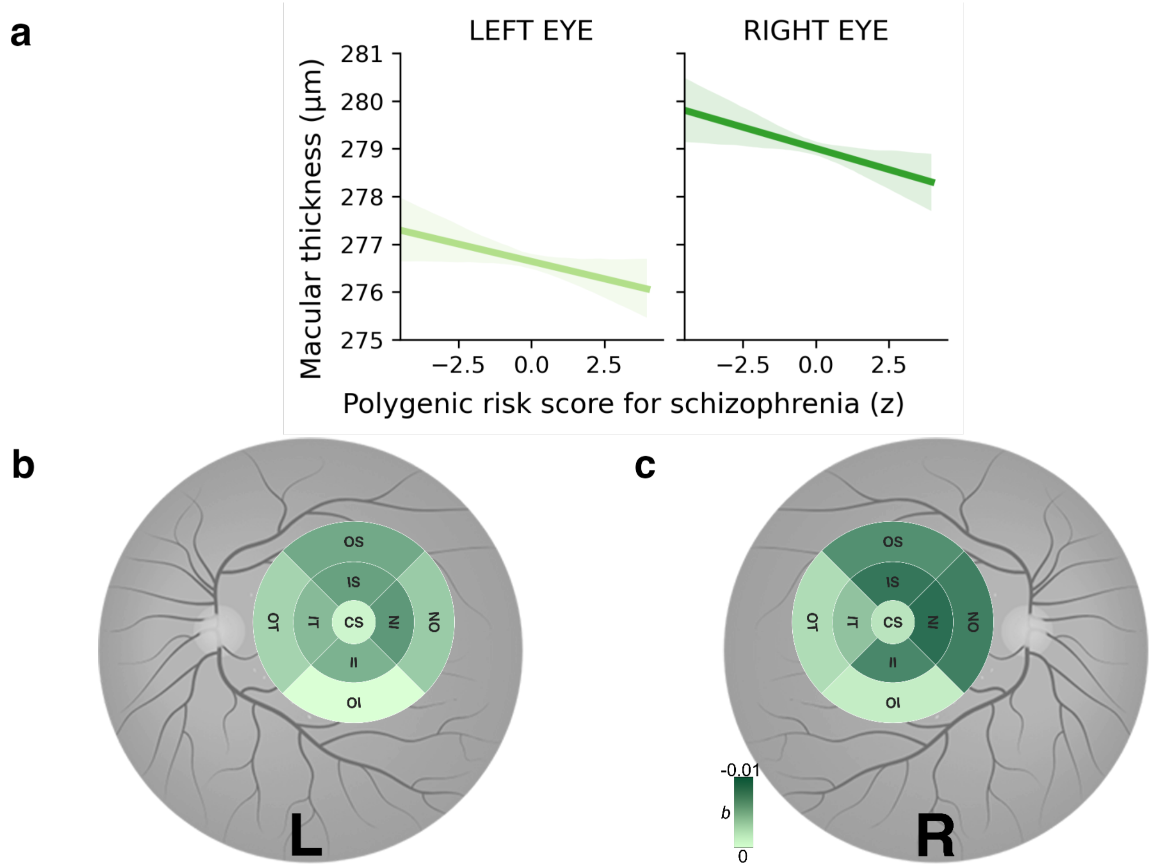

Figure S1. a. Association between polygenic risk score for schizophrenia and overall macular thickness in each eye respectively. Solid lines represent the regression estimates, while complementary shaded areas correspond to 95 percent confidence intervals. Individual macular subfields associated with polygenic risk scores for schizophrenia for both left eye (b) and right eye (c). Color coding corresponds to coefficients from robust linear regression model. Higher b values correspond to thinner macular subfields with increasing polygenic risk scores for schizophrenia. The sizes of subfields are slightly inflated for visualization purposes only. CS: Center subfield, IS: Inner Superior, OS: Outer Superior, IN: Inner Nasal, ON: Outer Nasal, II: Inner Inferior, OI: Outer Inferior, IT: Inner Temporal, OT: Outer Temporal.

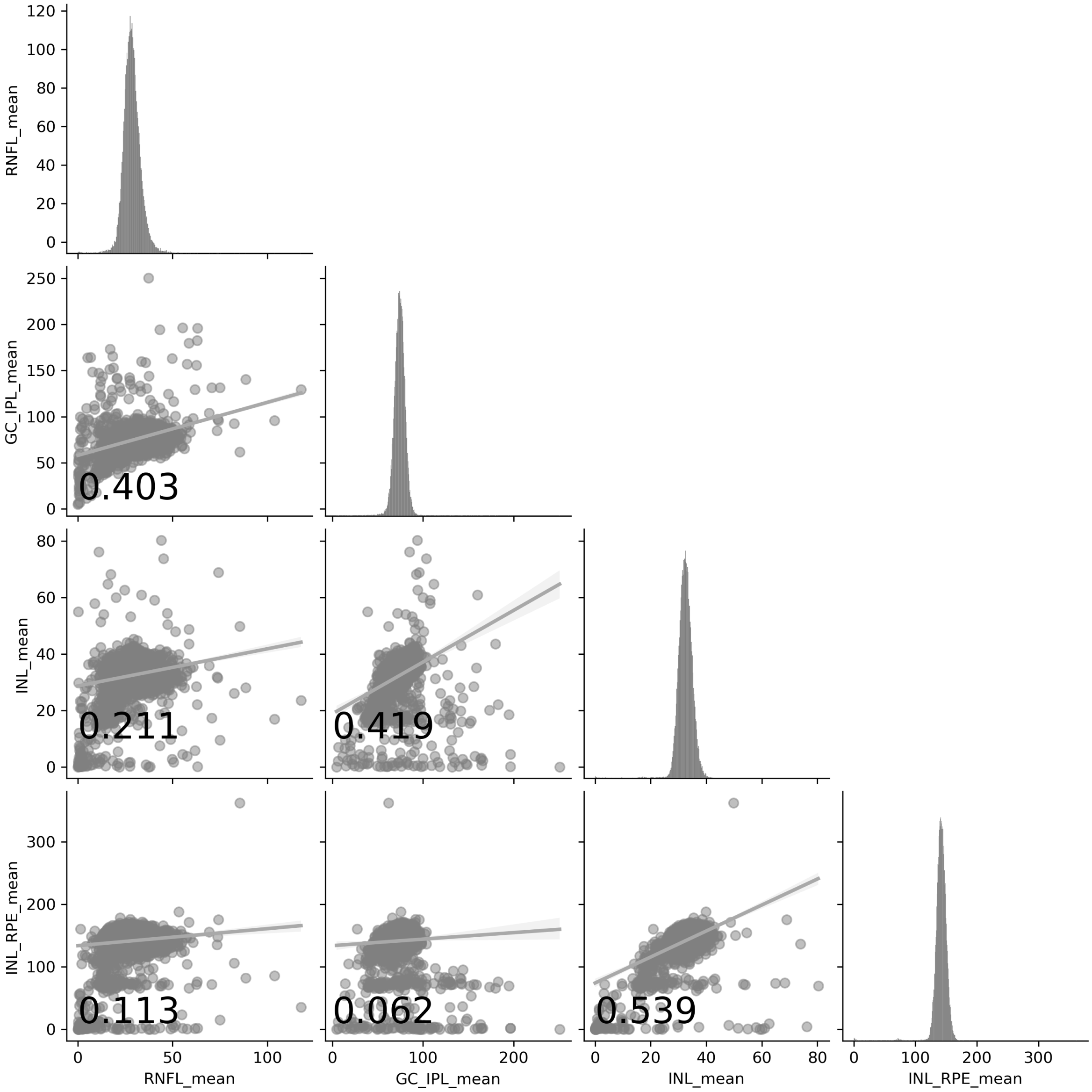

Figure S2. Pearson correlation coefficients between retinal phenotypes and showing their individual distribution.

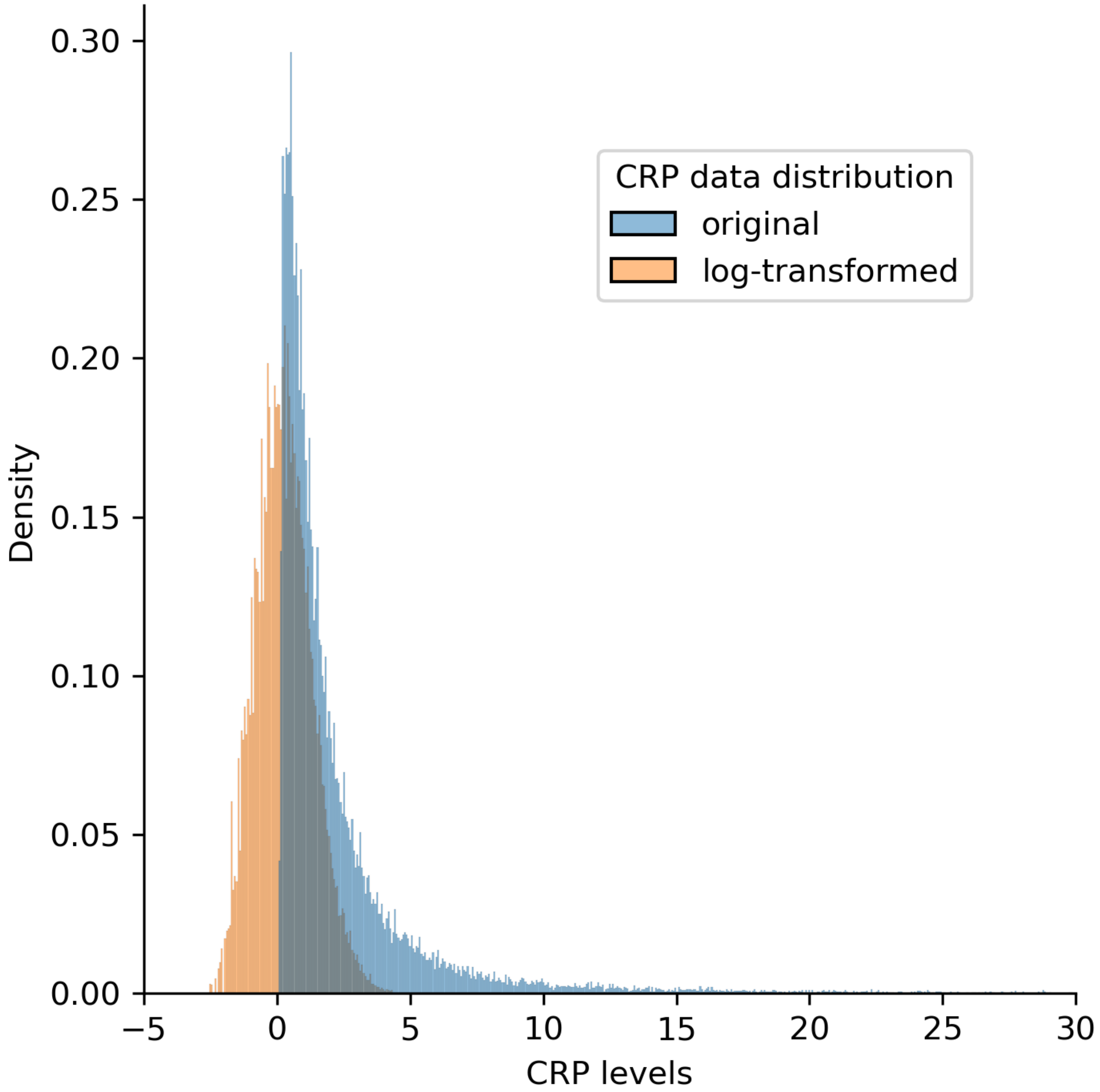

Figure S3. Logarithmic transformation of CRP measurements
