# Supplementary material for: Genetic susceptibility to schizophrenia through neuroinflammatory pathways associated with retinal thinness
Source: Nat Ment Health. 2025 Apr 21;3(5):538–47. doi: 10.1038/s44220-025-00414-6 (PMC12066354; doi:10.1038/s44220-025-00414-6)
Supplement: Supplementary file 2 — Reporting Summary [file 44220_2025_414_MOESM2_ESM.pdf]

nature portfolio

Corresponding author(s): Prof. Philipp Homan

Last updated by author(s): Mar 7, 2025

Reporting Summary

Nature Portfolio wishes to improve the reproducibility of the work that we publish. This form provides structure for consistency and transparency in reporting. For further information on Nature Portfolio policies, see our Editorial Policies and the Editorial Policy Checklist.

Statistics

For all statistical analyses, confirm that the following items are present in the figure legend, table legend, main text, or Methods section.

n/a	Confirmed	
☐	☒	The exact sample size (*n*) for each experimental group/condition, given as a discrete number and unit of measurement
☐	☒	A statement on whether measurements were taken from distinct samples or whether the same sample was measured repeatedly
☐	☒	The statistical test(s) used AND whether they are one- or two-sided<br>*Only common tests should be described solely by name; describe more complex techniques in the Methods section.*
☐	☒	A description of all covariates tested
☐	☒	A description of any assumptions or corrections, such as tests of normality and adjustment for multiple comparisons
☐	☒	A full description of the statistical parameters including central tendency (e.g. means) or other basic estimates (e.g. regression coefficient) AND variation (e.g. standard deviation) or associated estimates of uncertainty (e.g. confidence intervals)
☐	☒	For null hypothesis testing, the test statistic (e.g. *F*, *t*, *r*) with confidence intervals, effect sizes, degrees of freedom and *P* value noted<br>*Give P values as exact values whenever suitable.*
☒	☐	For Bayesian analysis, information on the choice of priors and Markov chain Monte Carlo settings
☒	☐	For hierarchical and complex designs, identification of the appropriate level for tests and full reporting of outcomes
☐	☒	Estimates of effect sizes (e.g. Cohen's *d*, Pearson's *r*), indicating how they were calculated

Our web collection on statistics for biologists contains articles on many of the points above.

Software and code

Policy information about availability of computer code

Data collection	No software was used for data collection in this study.
Data analysis	All data analyses were performed between June 2023 and February 2024 using Python (version 3.9.12) and R (version 4.0.5). The polygenic risk scores were computed using PRSet (version 2.3.5)14 . The manuscript was produced with the R packages rmarkdown (version 2.21)49; MASS (version 7.3.58.4)50; sjPlot (version 2.8.14)51; represearch (version 0.9; https://github.com/ phoman/represearch/); kableExtra (version 1.3.4)52; knitr (1.43)53; papaja (version 0.1.1)54; matplotlib (version 3.7.3)55; seaborn (version 0.11.2)56; and scipy (version 1.9.1).57 All code will be made freely available after publication to ensure reproducibility at https://github.com/homanlab/prsoct/.

For manuscripts utilizing custom algorithms or software that are central to the research but not yet described in published literature, software must be made available to editors and reviewers. We strongly encourage code deposition in a community repository (e.g. GitHub). See the Nature Portfolio guidelines for submitting code & software for further information.

Data

Policy information about availability of data

All manuscripts must include a data availability statement. This statement should provide the following information, where applicable:

- Accession codes, unique identifiers, or web links for publicly available datasets
- A description of any restrictions on data availability
- For clinical datasets or third party data, please ensure that the statement adheres to our policy

The summary statistics for the schizophrenia GWAS are publicly available at https://doi.org/10.6084/m9.figshare.19426775.v6, while the discovery data used in the study is based on the GRCh37/hg19 human genome assembly, which can be accessed through the NCBI Datasets resource at https://www.ncbi.nlm.nih.gov/datasets/genome/GCF_000001405.13/.
The rest of the data utilized in this study is publicly available at the UK Biobank (http://www.ukbiobank.ac.uk/) and was accessed via application number 102266.

Human research participants

Policy information about studies involving human research participants and Sex and Gender in Research.

Reporting on sex and gender	Sex as determined from genotyping analysis
Population characteristics	The covariate-relevant population characteristics of the human research participants in the study are as follows:<br>Sample Size: The study included 34,939 individuals out of 64,283 initially recruited participants.<br>Gender Distribution: 19,070 (54.58%) were female, and 15,869 (45.42%) were male.<br>Diagnoses: Individuals with diabetes mellitus and hypertension were 19,440 and 5204, respectively.<br>Age: The mean age of participants was 56.87 years with a standard deviation of 7.99 years.<br>Body Mass Index (BMI): The mean BMI was 27.23 with a standard deviation of 4.67.<br>Smoking Status:<br>Current smokers: 3,374 individuals<br>Previous smokers: 12,644 individuals<br>Non-smokers: 18,921 individuals<br>Drinker Status:<br>Current drinker: 32,810 individuals<br>Previous drinker: 1,108 individuals<br>Non-drinker: 1,021 individuals<br>Socioeconomic Status: Measured by the Townsend Deprivation Index, the mean score was -1.36 with a standard deviation of 2.81.<br>Genotypic Information: Participants were genotyped using the Applied Biosystems UK BiLEVE Axiom Array and the Applied Biosystems UK Biobank Axiom Array. Quality control measures included retaining SNPs with minor allele frequency >1% and INFO score >0.8, and excluding ambiguous SNPs.
Recruitment	UK Biobank recruited 500,000 people across the country aged between 40 and 69 years from 2006 to 2010. Assessments were undertaken in 22 centres in Scotland, England and Wales. There were five parts to the UK Biobank assessment process, which lasted between 2-3 hours. For more details, see https://www.ukbiobank.ac.uk/enable-your-research/about-our-data/baseline-assessment.
Ethics oversight	UK Biobank has approval from the North West Multi-centre Research Ethics Committee (MREC) as a Research Tissue Bank (RTB) approval. This approval means that researchers do not require separate ethical clearance and can operate under the RTB approval.

Note that full information on the approval of the study protocol must also be provided in the manuscript.

Field-specific reporting

Please select the one below that is the best fit for your research. If you are not sure, read the appropriate sections before making your selection.

☒ Life sciences ☐ Behavioural & social sciences ☐ Ecological, evolutionary & environmental sciences

For a reference copy of the document with all sections, see nature.com/documents/nr-reporting-summary-flat.pdf

Life sciences study design

All studies must disclose on these points even when the disclosure is negative.

Sample size	The study's use of a large, well-characterized cohort with a final sample size of 34,000 UK Biobank participants provides robust statistical power to detect even modest associations, making additional power analysis unnecessary.
Data exclusions	Data underwent a SNP-level QC (MAF < 0.005 and INFO score < 0.4) and sample-level QC (retaining individuals with missing rate in autosomes

Data exclusions	=< 0.02, which were not outliers for genotype missing- ness or heterozygosity, not being genetically related to third-degree relatives, not being sex-discordant and of White British or Irish ethnicity according to genetic grouping). We also excluded all optical coherence tomography images with the worst 20% image quality and individuals with eye disorders and diseases known to affect the eye, including diabetes related eye diseases, glaucoma, macular degeneration, injury or trauma resulting in loss of vision. We also excluded individuals with highly myopic and hyperopic eyes, individuals with a ICD-10 diagnosis (F20 to F29), those who were medicated with antipsychotics.
Replication	All association analyses and generation of the manuscript are automated and can be replicated with a single click of button.
Randomization	The study is an observational analysis using existing data from the UK Biobank. Observational studies typically do not involve interventions or manipulations of variables, so randomization is not applicable.
Blinding	Blinding did not apply to this observational study.

Reporting for specific materials, systems and methods

We require information from authors about some types of materials, experimental systems and methods used in many studies. Here, indicate whether each material, system or method listed is relevant to your study. If you are not sure if a list item applies to your research, read the appropriate section before selecting a response.

Materials & experimental systems

n/a	Involved in the study
☒	☐ Antibodies
☒	☐ Eukaryotic cell lines
☒	☐ Palaeontology and archaeology
☒	☐ Animals and other organisms
☒	☐ Clinical data
☒	☐ Dual use research of concern

Methods

n/a	Involved in the study
☒	☐ ChIP-seq
☒	☐ Flow cytometry
☒	☐ MRI-based neuroimaging
